# Comprehensive Analysis of *blaCTX-M1* Gene Expression Alongside *iutA*, *csgA*, and *kpsMII* Virulence Genes in Septicemic *Escherichia coli* Using Real-Time PCR

**DOI:** 10.3390/microorganisms13010095

**Published:** 2025-01-06

**Authors:** Mohsen Karbalaei, Mojgan Esmailpour, Valentyn Oksenych, Majid Eslami

**Affiliations:** 1 Department of Microbiology and Virology, School of Medicine, Jiroft University of Medical Sciences, Jiroft 78616-34200, Iran; mohsenkarbalaei691@gmail.com; 2 Medical Faculty, Shiraz University of Medical Sciences, Shiraz 71348-14336, Iran; 3 Department of Clinical and Molecular Medicine, Norwegian University of Science and Technology (NTNU), 7028 Trondheim, Norway; 4 Department of Biosciences and Nutrition, Karolinska Institutet, 14183 Huddinge, Sweden; 5 Department of Bacteriology and Virology, Semnan University of Medical Sciences, Semnan 35147-99442, Iran

**Keywords:** *blaCTX-M1*, *Escherichia coli*, real-time PCR, septicemic, virulence gene

## Abstract

**Background:** Sepsis is a serious worldwide health concern, and *Escherichia coli* (*E. coli*) is the main cause. This study investigates the co-expression of *blaCTX-M1* and *iutA, csgA*, and *kpsMII* genes in *E. coli* isolated from septicemic patients, aiming to clarify the interaction between virulence and resistance. **Methods:** This study evaluated 100 *E. coli* isolates from septicemic patients. With the disc diffusion method, antibiotic susceptibility was confirmed. The use of ceftazidime–clavulanic acid allowed for the confirmation of ESBL. PCR and real-time PCR were used to detect virulence and beta-lactamase genes. The expression levels of important genes were compared between isolates in LB and blood. **Results:** Antibiotic resistance was common in isolates carrying *blaCTX-M1*, including tetracycline (93%) and erythromycin (99%). Instead, there was no resistance to fosfomycin and 3% resistance to carbapenems. Real-time PCR revealed more expression levels in blood for the virulence genes *kpsMII* and *csgA*. Pathogenicity and resistance increased with *blaCTX-M1* co-expression with the *kpsMII* and *csgA* genes. **Conclusions**: The coexistence of ESBL and virulence genes in *E. coli* isolates significantly increases antibiotic resistance and infection severity. Monitoring of these genes is critical for developing effective therapeutic strategies. The key to treating these diseases is having sophisticated diagnostic tools and using antibiotics cautiously.

## 1. Introduction

Two important aspects of bacterial infections that provide significant challenges to global healthcare are urinary tract infections (UTIs) and sepsis [1]. UTIs are among the most prevalent bacterial infections in the world and are primarily brought on by *Escherichia coli* (*E. coli*), especially uropathogenic strains (UPEC) [2,3]. A variety of virulence factors, including as adhesion structures, toxins, and biofilm-forming capacities, which facilitate colonization, invasion, and persistence in the urinary tract, characterize the pathogenesis of UPEC [4]. The therapeutic landscape has become even more complex due to the alarming growth in antibiotic resistance, particularly as a result of the emergence of extended-spectrum beta-lactamases (ESBLs) [5]. Resistance to various beta-lactam antibiotics including cephalosporins and penicillins is mediated by ESBLs, often prompting the use of carbapenems or other last-line therapeutic options. A major consequence of bloodstream infections, particularly those caused by *E. coli* which produces the ESBLs, is sepsis, a critical and possibly deadly disease that results from a dysregulated host immunological response to infection. Due to the high rates of morbidity and mortality associated with this disease, it is essential that adequate antibiotics be started as soon as possible in order to improve patient outcomes. Poor prognoses have frequently been linked to delayed or inadequate treatments, especially in severe cases involving multi-organ failure or septic shock [6,7].

The growing concern about *Enterobacteriaceae* strains that carry ESBL genes, like *blaCTX-M1, blaTEM*, and *blaSHV*, has been highlighted by recent investigations. Because these genes enable the bacteria to hydrolyze a variety of beta-lactam antibiotics, such as cephalosporins and monobactams, they play a crucial role in antimicrobial resistance. Moreover, plasmids, which usually include virulence factors, are often the location of these resistance genes [8]. These strains may become greater risks as a result of the co-expression of virulence and resistance factors, making treatment and control more challenging. Adhesins, siderophores, and toxins are examples of virulence factors that are essential to the bacteria’s capacity to colonize host tissues, evade the immune system, and cause tissue damage. These elements play a significant role in the systemic spread of the bacteria and cause diseases. Since these pathogens can survive and multiply in hostile environments, they cause prolonged infections, especially during antibiotic treatment. Therefore, increasing in the phenomenon of antibiotic resistance, in turn, complicates treatment strategies and presents important challenges for clinical management [9].

The prevalence of ESBL-producing UPEC strains has been observed to vary by region, with higher rates reported in hospital settings and among vulnerable populations, including children, the elderly, and immunocompromised individuals. Infections caused by these resistant strains are often more difficult to treat, resulting in prolonged hospital stays, increased healthcare costs, and higher mortality rates [10]. This problem is made severe by limitations of effective antibiotics to treat these infections, especially when carbapenems, which are frequently used as last-resort antibiotics, are also made ineffective by the formation of carbapenemase. One of the major challenges to treating infections caused by *E. coli* is antibiotic resistance [11]. It can rapidly become resistant to different antibiotics because of its capacity to create a variety of toxins and virulence factors. Virulence genes such as *cdt, kpsMII, iutA, hlyA*, and *cnf1* are among the most important virulence genes that are critical to identify and genotype. These genes increase the bacterium’s pathogenic potential and aid in the propagation of antibiotic resistance in bacterial populations, in addition to helping the bacterium evade the human immune system [12,13].

In this study, the major purpose is to comprehensively investigate the expression of key genes related with both antibiotic resistance and pathogenicity in *E. coli* isolates from septicemic patients. Along with virulence genes like *iutA, csgA*, and *kpsMII*, the emphasis is on the *blaCTX-M1* gene, which is essential for the generation of ESBL. Comprehending the co-expression of these genes is essential because it provides insight into how sepsis-causing *E. coli* strains not only withstand antibiotic therapy but also improve their capacity to colonize host tissues and evade immune responses.

## 2. Material and Methods

### 2.1. Sample Collection

A total of 100 *E. coli* isolates were obtained from patients with septicemia hospitalized in Semnan, Iran, during January 2021 to February 2023. Then, in order to store them for a longer period, we used a tryptic soy broth consisting of 20% glycerol (Merck, Darmstadt, Germany); isolates were stored at a temperature of −20 °C.

### 2.2. Antibiotic Susceptibility Assessment

The antibiotic susceptibility of the isolates was evaluated against various antibiotics using the Kirby–Bauer disc diffusion method according to the Clinical and Laboratory Standards Institute (CLIS) guidelines (2023). The antibiotic panel used in the disc agar diffusion method included the following antibiotics with their respective disc concentrations: ceftazidime (CAZ: 30 μg), cefotaxime (CTX: 30 μg), erythromycin (ERY: 15 μg), gentamicin (GM: 10 μg), tetracycline (TE: 30 μg), trimethoprim-sulfamethoxazole (TS: 25 μg), amoxicillin-clavulanic acid (AMC: 10/20 μg), amoxicillin (AMX: 30 μg), imipenem (IMI: 10 μg), ciprofloxacin (CIP: 5 μg), piperacillin-tazobactam (PTZ: 10/100 μg), meropenem (MEN: 10 μg), cefazolin (CZ: 30 μg), nitrofurantoin (FN: 300 μg), and fosfomycin (FO: 200 μg)

### 2.3. Detection of ESBL-Positive Isolates

Screening for ESBL production was carried out using a 30 μg ceftazidime (CAZ) disc using the disc diffusion method according to CLSI guidelines (CLSI 2023). Isolates that showed a zone of inhibition < 22 mm were considered as non-susceptible to the CAZ disc and therefore potential ESBL producers. Then, a CAZ disc (30 μg) and a combined disc of CAZ with clavulanic acid (CAC) (30/10 μg) were used as a confirmatory test for each ESBL-possible isolate. Both of the discs were placed on a cultured Muller–Hinton agar (MHA) plate and incubated overnight at 37 °C. An increase of ≥5 in zone diameter was considered phenotypically *blaCTX-M1* beta-lactamase positive.

### 2.4. Detection of Beta-Lactamase Genes

The optimized primers were used to amplify the genes such as *blaTEM*, *blaCTX-M1*, *blaSHV*, *blaKPC*, *blaIPM*, *blaVIM*, and *blaOXA-48*. Bacterial DNA was prepared by suspending one or two fresh colonies in 50 μL of sterile distilled water and heating at 95 °C for 5 min. PCR amplifications of sequences were performed using 30 amplification cycles with this program: 30 s at 94 °C (denaturation), 90 s at 72 °C (elongation), and final elongation at 72 °C for 5 min; in the annealing stage, time and temperature were specific for each primer pair and are listed in Table 1.

### 2.5. Genotyping of Virulence Genes

*E. coli* isolates were tested for the presence of virulence genes including *cdt* (cytolethal distending toxin), *kpsMII* (capsular polysaccharide synthesis K1), *tcpC* (receptor Toll/interleukin 1), *iutA* (iron uptake/transport), *traT* (serum survival), *hlyA* (α-haemolysin), *cnf1* (cytotoxic necrotizing factor 1), *ibeA* (invasion of brain endothelium), *vat* (vaculating autoinducer toxin), *sat* (secretion autoinducer toxin), *pic* (serine protease autoinducer), and *csgA* (curli fimbriae) (Table 1).

### 2.6. Real-Time PCR

In this step, first, isolates were cultured in both LB and blood culture media. After 2 h, using the SYBR-Green real-time PCR method, isolates obtained from both media were examined for the gene expression level. We compared the expression of *blaCTX-M1* with virulence genes, e.g., *iutA*, *csgA*, and *kpsMII*. *gyrB* was used as an internal control (Table 1).

### 2.7. Ethical Approval

This study was approved by the ethical committee of Semnan University of Medical Sciences Semnan, Iran, with the following ethical ID: 1584, IRCT: IR.SEMUMS. REC.1398.28.

## 3. Results 

### 3.1. Sample Processing and Phenotypic Characterization

During the study period, a total of 100 *E. coli* isolates were collected from patients who had been hospitalized due to septicemia in Semnan, Iran. Immediately after collection, all samples were processed, and lactose-fermenting colonies with appropriate colony morphology were presumptively identified as *E. coli*, and further confirmation was carried out using standard conventional biochemical tests. In total, 65% of the isolates were related to the blood samples, which included the highest percentage of clinical isolates.

### 3.2. Antibiotic Susceptibility Profile

Following the CLSI interpretive criteria, the collected isolates showed phenotypic resistance/intermediate rates to erythromycin (99%), amoxicillin (93%), tetracycline (93%), and cefazolin (75%). Relatively low resistance, about 3%, was observed for imipenem and meropenem, while no resistance was seen for fosfomycin. On the other hand, resistance to piperacillin/tazobactam and nitrofurantoin was reported at about 7% and 9%, respectively. More than 50% of *E. coli* isolates‏ ‏were resistant‏ ‏to other antibiotics. In the present study, we observed a high resistance rate (45%) against co-amoxiclav. Nevertheless, all isolates (100%) were resistant to fosfomycin, and the rate of resistance to imipenem/meropenem was 97% (Table 2).

### 3.3. The Prevalence of ESBL Genes

**Figure 1:**
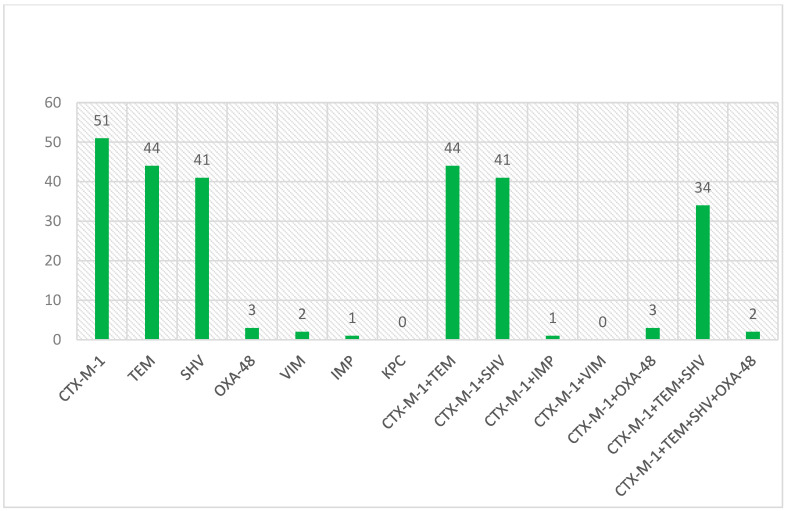
The occurrence patterns of genes encoding ESBL enzyme in investigated isolates.

All isolates were screened for *blaCTX-M1*, *blaTEM*, and *blaSHV* encoding genes. The occurrence patterns of ESBL genes in studied isolates are shown in Figure 1.

Based on the antibiotic susceptibility results, there was a high resistance rate in isolates containing *blaTEM* gene for amoxicillin (100%), cefazolin (100%), cefotaxime (100%), erythromycin (97.7%), and ceftazidime (91%), whereas a relatively low resistance (2.2%) was observed to imipenem, meropenem, and nitrofurantoin. In addition, no resistance was found to fosfomycin. Except for five antibiotics, fosfomycin, nitrofurantoin, Piperacillin-tazobactam, imipenem, and meropenem, for the other studied antibiotics, more than 55% resistance was observed in isolates with the *blaTEM* gene. In isolates with the *blaCTX-M1* gene, a high rate of resistance was observed for amoxicillin, cefazolin and cefotaxime (100%), erythromycin (98%), and ceftazidime (93%), but low resistance rates were found for imipenem (2%), meropenem (2%), piperacillin-tazobactam (4%), and nitrofurantoin (4%). In addition, no resistance to fosfomycin antibiotic was observed. In isolates with the *blaSHV* gene, a high rate of resistance was observed for amoxicillin (100%), cefazolin (100%), cefotaxime (100%), erythromycin (98%), and ceftazidime (93%), whereas the lowest resistance rates were found for imipenem, meropenem, and piperacillin-tazobactam (3%). In addition, no resistance to the fosfomycin antibiotic was observed. The values of antibiotic resistance associated with ESBL isolates of *E. coli* are shown in Table 3.

**Figure 2:**
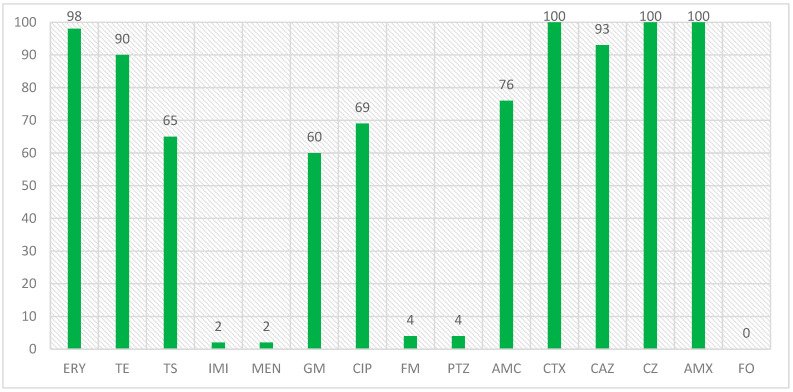
Antibiotic resistance percentage in isolates with *blaCTX-M1* gene.

In a study that was conducted on the isolates with the *blaCTX-M1* beta-lactamase gene and the antibiotic resistance of these isolates, the results were that the highest number of resistance was related to amoxicillin, cefazolin, and cefotaxime with 100% resistance, erythromycin with 98% resistance and ceftazidime with 93% resistance, and the lowest number of resistance was related to imipenem and meropenem, piperacillin/tazobactam and nitrofurantoin with 2 and 4%, respectively, and no resistance was observed to the fosfomycin antibiotic in the isolated *E. coli* isolates. The percentage of antibiotic resistance in isolates with the *blaCTX-M1* gene is also shown in Figure 2.

### 3.4. Detected Virulence Genes in ESBL-Producing Isolates

**Figure 3:**
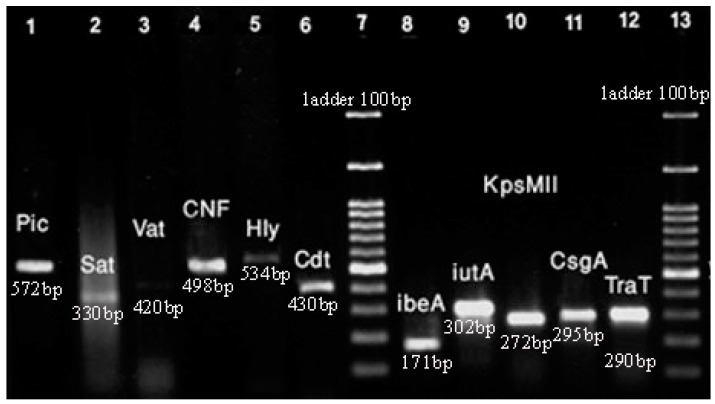
PCR for virulence genes of *E. coli* isolates. Lane 1: *pic* (572 bp); Lane 2: *sat* (330 bp); Lane 3: *vat* (420 bp); Lane 4: *cnf1* (498 bp); Lane 5: *hlyA* (534); Lane 6: *cdt* (430 bp); Lane 7: ladder; Lane 8: *ibeA* (171 bp); Lane 9: *iutA* (302 bp); Lane 10: *kpsMII* (272 bp); Lane 11: *csgA* (295 bp); Lane 12: *traT* (290 bp); Lane 13: ladder. Note: amplification associated with *tcpC* gene was not sufficient for detection in PCR.

As mentioned above, we investigated virulence genes such as *cdt*, *kpsMII*, *tcpC*, *iutA*, *traT*, *hlyA*, *cnf1*, *ibeA*, *vat*, *sat*, *pic*, and *csgA*. The frequency of virulence genes in the clinical samples of *E. coli* has been shown in Figure 3.

The *hlyA* virulence gene was the most frequent gene among the isolates with 86%. Also, the frequency of other genes such as *csgA* and *traT* was 84% and 79%, respectively. Based on the results, 50% of isolates carried 35 virulence genes. The lowest frequency was related to the *pic* and *cdt* genes, with 8% and 5% of isolates, respectively.

### 3.5. Real-Time PCR

**Figure 4:**
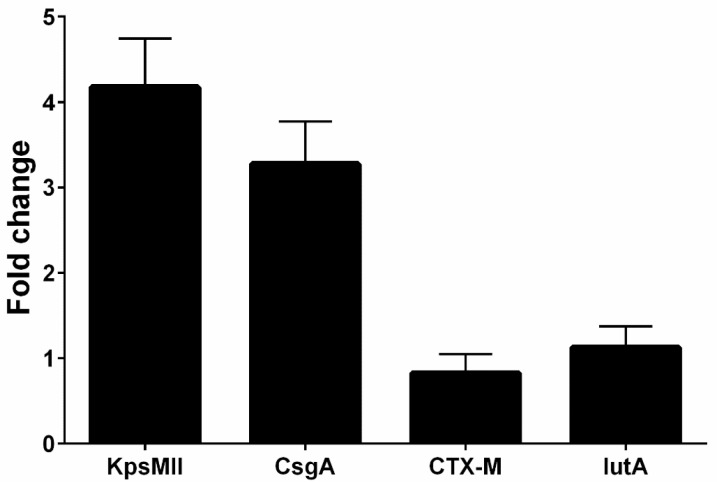
The fold change of the genes (*blaCTX-M1*, *KPSMII*, *CsgA* and *IutA*) in real-time PCR.

**Figure 5:**
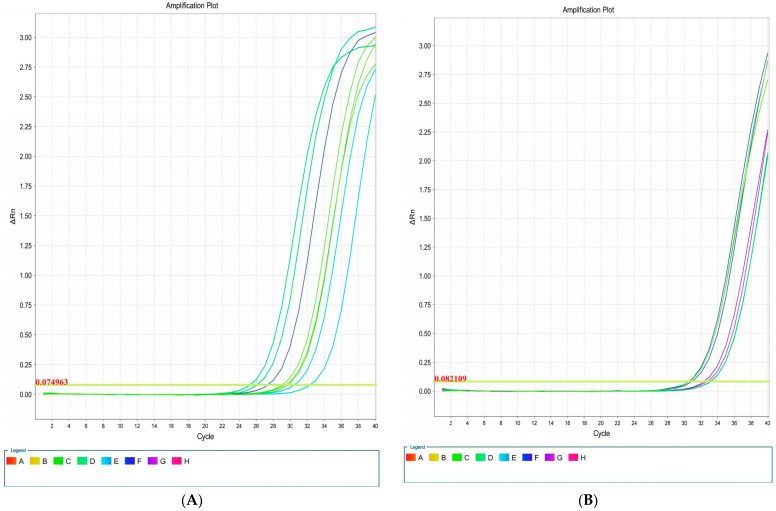

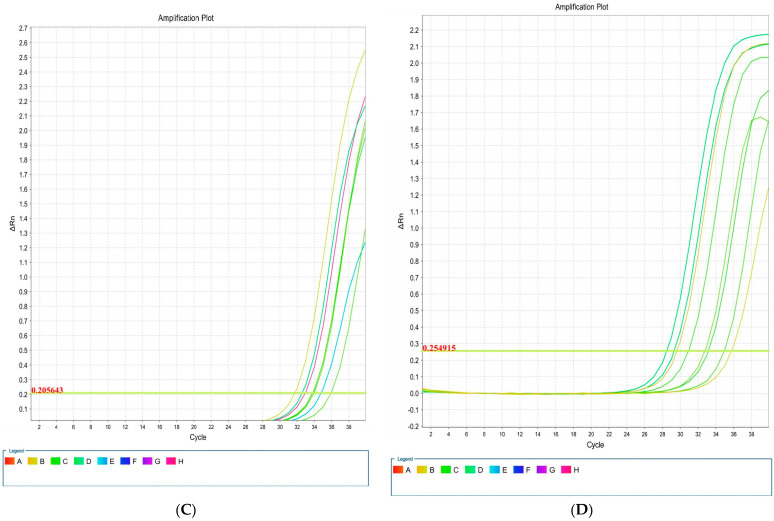
Amplification plots associated with *E. coli* genes: *csgA* (**A**), *blaCTX*-M1 (**B**), *iutA* (**C**), and *kpsMII* (**D**) in real-time PCR.

Overall, four isolates had *blaCTX-M1*, *iutA*, *csgA*, and *kpsMII* genes, in both LB and blood media. Then, RNA extraction and cDNA synthesis was performed, and real-time PCR was carried out. Real-time results showed that the isolates had the highest *kpsMII* gene expression. The fold change of the results of the real-time PCR is shown in Figure 4. The level of expression of *csgA* and *kpsMII* genes in blood culture compared with LB culture was four- and three-fold, respectively (Figure 5).

The results of the real-time PCR analysis indicated that the *KpsMII* gene exhibited the highest expression level, with its expression in blood culture being approximately four-fold higher than in LB medium. Following this, the *csgA* gene showed about a three-fold increase in expression in the blood-enriched medium compared to the LB medium. In contrast, the expression levels of the *blaCTX*-M and *iutA* genes demonstrated no significant differences between the LB and blood-enriched media.

In our study of *E. coli* isolates, focusing on the expression of four target genes through real-time PCR, it was observed that the *blaCTX*-M and iutA genes had the lowest expression levels. Furthermore, our investigation specifically targeting the *KpsMII* gene in strains harbouring this gene revealed that its expression was markedly higher compared to the other three genes, namely *csgA*, *iutA*, and *blaCTX*-M. These findings underscore the pivotal role of the *KpsMII* gene in the pathogenesis of bloodstream infections caused by *E. coli.*

## 4. Discussion

The global distribution of ESBL-producing *Enterobacteriaceae* underscores significant regional disparities, with prevalence rates notably higher in Asia, South America, and Africa compared to North America and Europe [29]. These differences can be attributed to multiple factors, including the misuse and overuse of antimicrobials, suboptimal hygiene standards, the availability of counterfeit drugs, and the burden of infectious diseases in resource-limited settings [30,31]. Additionally, the lack of advanced diagnostic capabilities in many regions exacerbates the challenge by delaying appropriate treatment and containment measures. The frequent use of third-generation cephalosporins, such as ceftriaxone and cefotaxime, in empirical therapies is directly related to the widespread resistance to penicillins and cephalosporins [32]. Particularly in hospital settings, where horizontal gene transfer via plasmids enables rapid distribution of ESBL genes across many bacterial species, this approach has helped in the development and spread of resistant strains. The increased exposure to resistant microorganisms in healthcare settings makes hospitalization itself a significant risk factor [33]. Cross-resistance to other widely used antibiotics, such as aminoglycosides, tetracyclines, and trimethoprim-sulfamethoxazole, further complicates the problem and significantly reduces the alternatives for empirical therapy. These results demonstrate the urgent necessity for international measures to stop the spread of *Enterobacteriaceae* that produce ESBLs. To address the causes of antimicrobial resistance completely, efforts must include careful application of antibiotics, improving infection control protocols, enhancing diagnostic capacities, and increasing international collaboration [34].

Sepsis is considered a life-threatening problem that can be caused by a broad range of pathogens, including Gram-negative and Gram-positive bacteria, fungi, and viruses. This study provides a comprehensive investigation of the relationship between the expression of virulence genes in *E. coli* isolates from sepsis patients and antibiotic resistance induced by beta-lactamase genes. The results demonstrate how common beta-lactamase genes, such as *blaCTX-M1*, *blaTEM*, and *blaSHV*, are and how they play a significant role in antibiotic resistance. These genes are essential for decreasing the effectiveness of beta-lactam antibiotics such as cefazolin, amoxicillin, and cefotaxime. Notably, resistance rates exceeding 90% in these isolates emphasize the urgent need for alternative therapeutic strategies and severe antibiotic supervision. Conversely, the low resistance observed against carbapenems such as imipenem and meropenem, reported in only 2% of isolates, offers hope for their use as effective therapeutic options. However, the potential emergence of resistance to these drugs necessitates careful monitoring. Beta-lactamase genes like *blaCTX-M1* enhance bacterial survival under therapeutic pressure by enabling extensive hydrolysis of beta-lactam antibiotics. The coexistence of *blaTEM* and *blaSHV* with virulence genes like *kpsMII*, *iutA*, and *csgA* highlights the intricate relationship between bacterial virulence and antibiotic resistance, which increases pathogenicity and bacterial survival.

Regarding virulence gene expression, real-time PCR results revealed elevated expression of genes like *kpsMII* and *csgA* in the bloodstream. The *kpsMII* gene, essential for polysaccharide capsule synthesis, facilitates evasion from the host immune system by inhibiting phagocytosis, thereby promoting systemic bacterial survival and dissemination. Additionally, the expression of *csgA*, which aids biofilm formation, enhances bacterial resistance to antibiotics and predisposes to chronic infections. Different functions in pathogenesis are also played by other virulence genes. For example, hemolysin is produced by the *hlyA* gene, which has been found in 86% of isolates, and causes tissue damage and the spread of infections. Additionally, the *traT* gene, which is present in around 79% of isolates, suppresses the immune system, especially the complement system. These genes’ cohabitation with beta-lactamase genes demonstrates *E. coli*’s adaptive ability to combine increased virulence with high drug resistance.

The co-expression of virulence genes and antibiotic resistance genes in *E. coli* represents a significant adaptive mechanism that enhances bacterial survival in hostile host environments, particularly during infection and antibiotic treatment. This co-expression enables the pathogen to not only evade the host immune system but also resist the effects of therapeutic agents, thereby facilitating persistent infections and complicating treatment strategies. The capacity of the bacteria to survive in the circulation and tissues is largely dependent on the *kpsMII* and *csgA* genes, which are in charge of capsule production and biofilm formation, respectively. The polysaccharide capsule that *kpsMII* produces improves the pathogen’s capacity to evade the immune system and protects *E. coli* from host immunological responses like phagocytosis. Biofilm production driven by *csgA* improves this defence by protecting pathogens from immunological responses and increasing drug resistance. Biofilms serve as physical barriers that prevent antimicrobial medicines from penetrating, resulting in a persistent bacterial reservoir that can re-infect patients when therapy is finished. Concurrently, *E. coli* may neutralize antibiotics like cephalosporins and penicillins by using resistance genes like the *bla* genes, which encode beta-lactamases. These antibiotics lose their effectiveness when their beta-lactam ring is broken down by the formation of beta-lactamases. A synergistic impact is suggested by the coexistence of bla genes and virulence factors such as *hlyA* and *traT*. The resistance genes protect the bacteria from the selection pressure of antibiotic therapy, allowing for continued infection and bacterial persistence, while the virulence genes ensure the pathogen’s survival and growth within the host. Since infections caused by these multidrug-resistant strains are challenging to treat in clinical settings, the co-expression of virulence and resistance factors is especially problematic. Increased pathogenicity and resistance to widely used antibiotics complicate the development of effective therapeutic approaches, requiring the investigation of novel therapeutic approaches or approaches that can simultaneously target resistance and virulence mechanisms.

*E. coli* adapts to acidic environments by producing ATP primarily through glycolysis. Antibiotic-resistant strains, like ampP strepC, show increased tolerance to lactic acid (LA), a common antimicrobial. Efflux pump inhibition significantly reduces LA tolerance, linking antibiotic resistance to enhanced survival under LA stress [35,36,37]. Alpha-lipoic acid (LA) enhances the antibacterial activity of antimicrobial peptides (AMPs) and antibiotics by improving stability and membrane interaction. Its hydrophobic properties enable LA-modified AMPs, such as LA-Bac8c, to disrupt bacterial membranes through lipophilic and electrostatic interactions, causing depolarization, permeabilization, and cellular leakage. LA’s structural role amplifies AMP efficacy, targeting biofilms and overcoming bacterial resistance mechanisms. This synergy offers a promising approach to addressing antibiotic resistance and biofilm-related infections. Furthermore, LA’s structural and metabolic characteristics have a major role in combating bacterial resistance, providing a viable strategy against antibiotic-resistant pathogens and diseases linked to biofilms. These results highlight the therapeutic potential of drugs changed by LA in treating the worldwide problem of antimicrobial resistance [38,39,40].

This study demonstrates that the presence of beta-lactamase genes not only reinforces bacterial resistance to antibiotics but also, in conjunction with virulence gene expression, exacerbates infection severity. These findings emphasize the critical importance of detecting and monitoring resistance and virulence genes to facilitate the prevention and treatment of infections caused by this pathogen. Further research focusing on regulatory pathways governing these genes is essential for developing targeted therapeutic strategies.

## 5. Conclusions

This study offers an extensive analysis of the relationship between virulence gene expression and antibiotic resistance in *E. coli* isolates from patients with septicemia. With resistance rates over 90%, the results demonstrate the important role that beta-lactamase genes like *blaCTX-M1*, *blaTEM*, and *blaSHV* play in conferring resistance to beta-lactam antibiotics, such as cefazolin, amoxicillin, and cefotaxime. The possibility of developing resistance highlights the necessity of cautious monitoring and careful application of these last-resort antibiotics, even if resistance to carbapenems such as imipenem and meropenem has been found to be low (2%). Increased expression of virulence genes, specifically *kpsMII* and *csgA*, which increase pathogenicity by promoting immune evasion and biofilm formation, respectively, was also discovered in this study. By encouraging bacterial survival and persistence in adverse conditions, these genes, when combined with antibiotic resistance factors, make treating infections more difficult. The high frequency of virulence genes like *traT* and *hlyA* emphasizes their role in immune system evasion and systemic infections. In order improve the treatment of infections caused by *E. coli*, this study highlights the vital significance of detection and monitoring virulence and resistance genes. Future research on the mechanisms and regulatory processes controlling these genes is essential for creating efficient treatment plans and reducing the prevalence of antibiotic resistance worldwide.

## Figures and Tables

**Table 1:** Primers and conditions used to detect beta-lactamase genes, virulence typing and real-time PCR.

Target Gene	Product	Sequence	Annealing	Ref.
*blaTEM*	850 bp	FP: 5′-ATGAGTATTCAACATTTCCG-3′	53 °C for 1 min	[14] [15]
		RP: 5′-CCAATGCTTAATCAGTGAGG-3′		
*blaCTX-M1*	850 bp	FP: 5′-GGTTAAAAAATCACTGCGTC-3′	53 °C for 45 s	
		RP: 5′-TTGGTGACGATTTTAGCCGC-3′		
*blaSHV*	230 bp	FP: 5′-AAGATCCACTATCGCCAGCAG-3′	56 °C for 50 s	
		RP: 5′-ATTCAGTTCCGTTTCCCAGCGG-3′		
*blaKPC*	900 bp	FP: 5′-TGTCACTGTATCGCCGTC-3′	52 °C for 1 min	[16]
		RP: 5′-CTCAGTGCTCTACAGAAAACC-3′		
*blaIPM*	587 bp	FP: 5′-GAAGGCGTTTATGTTCATAC-3′	50 °C for 45 s	[17]
		RP: 5′-GTACGTTTCAAGAGTGATGC-3′		
*blaVIM*	389 bp	FP: 5′-GTTTGGTCGCATATCGCAAC-3′	57 °C for 1 min	
		RP: 5′-AATGCGCAGCACCAGGATAG-3′		
*blaOXA-48*	438 bp	FP: 5′-GCGTGGTTAAGGATGAACAC-3′	56 °C for 1 min	
		RP: 5′-CATCAAGTTCAACCCAACCG-3′		
*cdt*	430 bp	FP: 5′-AAATCACCAAGAATCATCCAGTTA-3′	58 °C for 1 min	[18]
		RP: 5′-AAATCTCCTGCAATCATCCAGTTTA-3′		
*kpsMII*	272 bp	FP: 5′-GCGCATTTGCTGATACTGTTG-3′	50 °C for 45 s	[19]
		RP: 5′-CATCCAGACGATAAGCATGAGCA-3′		
*tcpC*	544 bp	FP: 5′-GAGTGGAAGGAGGTTGAGGC-3′	61 °C for 1 min	[20]
		RP: 5′-GCAGTGCCATTTTATCCGCC-3′		
*iutA*	302 bp	FP: 5′-GGCTGGACATCATGGGAACTGG-3′	55 °C for 1 min	[21]
		RP: 5′-CGTCGGGAACGGGTAGAATCG-3′		
*traT*	290 bp	FP: 5′-GGTGTGGTGCGATGAGCACAG-3′	62 °C for 50 s	[22]
		RP: 5′-CACGGTTCAGCCATCCCTGAG-3′		
*hlyA*	534 bp	FP: 5′- GCATCATCAAGCGTACGTTCC-3′	49 °C for 1 min	[23]
		RP: 5′- AATGAGCCAAGCTGGTTAAGCT-3′		
*cnf1*	498 bp	FP: 5′- AAGATGGAGTTTCCTATGCAGGAG-3′	59 °C for 45 s	
		RP: 5′- CATTCAGAGTCCTGCCCTCATTAT-3′		
*ibeA*	171 bp	FP: 5′- AGGCAGGTGTGCGCCGCGTAC-3′	60 °C for 1 min	
		RP: 5′-TGGTGCTCCGGCAAACCATGC-3′		
*vat*	420 bp	FP: 5′-AACGGTTGGTGGCAACAATCC-3′	62 °C for 50 s	[12]
		RP: 5′-AGCCCTGTAGAATGGCGAGTA-3′		
*sat*	330 bp	FP: 5′-TCAGAAGCTCAGCGAATCATTG-3′	55 °C for 1 min	
		RP: 5′-CCATTATCACCAGTAAAACGCACC-3′		
*pic*	572 bp	FP: 5′-ACTGGATCTTAAGGCTCAGGAT-3′	58 °C for 45 s	
		RP: 5′-GACTTAATGTCACTGTTCAGCG-3′		
*csgA*	295 bp	FP: 5′-GGCGGAAATGGTTCAGATGTTG-3′	52 °C for 1 min	[24]
		RP: 5′-CGTATTCATAAGCTTCTCCCGA-3′		
*blaCTX-M1*	49 bp	FP: 5′-TGGGGGATAAAACCGGCAG-3′	53 °C for 1 min	[25]
		RP: 5′-GCGATATCGTTGGTGGTGC-3′		
*iutA*	59 bp	FP: 5′-CGGTGGCGTACGCTATCAGT-3′	59 °C for 1 min	[26]
		RP: 5′-GCGCGTAGCCGATGAAAT-3′		
*csgA*	68 bp	FP: 5′-GCGGTAATGGTGCAGATGTTG-3′	60 °C for 1 min	[27]
		RP: 5′-GAAGCCACGTTGGGTCAGA-3′		
*kpsMII*	134 bp	FP: 5′-GCACTGCTTGAGACACTGATTTACG-3′	62 °C for 1 min	[28]
		RP: 5′-GAAAGAATGATTAACAAACTCCAGGAG-3′		
*gyrB*	254 bp	FP: 5′-GCAAGCCACGCAGTTTCTC-3′	61 °C for 1 min	[27]
		RP: 5′-GGAAGCCGACCTCTCTGATG-3′		

**Table 2:** Results of antimicrobial susceptibility test.

Antibiotic Disc	Resistant (%)	Intermediate (%)	Sensitive (%)
Erythromycin (15 µg)	81	18	1
Tetracycline (30 µg)	74	19	7
Cotrimoxazole (25 µg)	66	7	27
Imipenem (10 µg)	1	2	97
Meropenem (10 µg)	1	2	97
Gentamycin (120 µg)	44	17	39
Ciprofloxacin (5 µg)	58	7	35
Nitrofurantoin (300 µg)	7	0	93
Piperacillin-tazobactam (110 µg)	9	0	91
Co-amoxiclav (30 µg)	32	45	23
Ceftazidime (30 µg)	36	20	44
Cefotaxime (30 µg)	44	7	49
Cefazolin (30 µg)	75	8	17
Amoxicillin (10 µg)	80	13	7
Fosfomycin (200 µg)	0	0	100

**Table 3:** Results of antibiotic resistance pattern of *E. coli* isolates by ESBL.

Genes	Antibiotics														
	CAZ	IMI	MEN	CZ	AMX	FO	E	TE	PTZ	TS	GM	CIP	FM	AMC	CTX
*blaTEM*	91	2.2	2.2	100	100	0	97.7	88.6	4.5	65.9	56.8	68.1	2.2	75	100
*blaSHV*	93	3	3	100	100	0	98	91	3	61	64	66	5	76	100
*blaCTX-M1*	93	2	2	100	100	0	98	90	4	65	60	69	4	76	100

## Data Availability

The corresponding author will provide the datasets created during and/or analyzed during the current investigation upon reasonable request.

## References

[1] Öztürk R., Murt A. (2020). Epidemiology of urological infections: A global burden. World J. Urol..

[2] Karam M.R.A., Habibi M., Bouzari S. (2019). Urinary tract infection: Pathogenicity, antibiotic resistance and development of effective vaccines against Uropathogenic *Escherichia coli*. Mol. Immunol..

[3] López-Sampedro I., Hernández-Chico I., Gómez-Vicente E., Expósito-Ruiz M., Navarro-Marí J.M., Gutiérrez-Fernández J. (2023). Evolution of Antibiotic Resistance in *Escherichia coli* and Klebsiella pneumoniae from Urine Cultures. Arch. Esp. De Urol..

[4] Bunduki G.K., Heinz E., Phiri V.S., Noah P., Feasey N., Musaya J. (2021). Virulence factors and antimicrobial resistance of uropathogenic *Escherichia coli* (UPEC) isolated from urinary tract infections: A systematic review and meta-analysis. BMC Infect. Dis..

[5] De Angelis G., Del Giacomo P., Posteraro B., Sanguinetti M., Tumbarello M. (2020). Molecular mechanisms, epidemiology, and clinical importance of β-lactam resistance in Enterobacteriaceae. Int. J. Mol. Sci..

[6] Fröding I. (2019). Bloodstream Infections with ESBL-Producing Enterobacterales: Prediction, Rapid Diagnosis and Molecular Epidemiology.

[7] Yousefi B., Pakdel A., Hasanpour S., Abdolshahi A., Emadi A., Pahlevan D., Dadashpour M., Eslami M. (2023). CTX-M gene and presence of insertion elements in patients with septicemia caused by *Escherichia coli*. Rev. Res. Med. Microbiol..

[8] Hussain H.I., Aqib A.I., Seleem M.N., Shabbir M.A., Hao H., Iqbal Z., Kulyar M.F.-e.-A., Zaheer T., Li K. (2021). Genetic basis of molecular mechanisms in β-lactam resistant gram-negative bacteria. Microb. Pathog..

[9] Pontes J.o.G.d.M., Fernandes L.S., dos Santos R.V., Tasic L., Fill T.P. (2020). Virulence factors in the phytopathogen–host interactions: An overview. J. Agric. Food Chem..

[10] Butcher C.R., Rubin J., Mussio K., Riley L.W. (2019). Risk factors associated with community-acquired urinary tract infections caused by extended-spectrum β-lactamase-producing *Escherichia coli*: A systematic review. Curr. Epidemiol. Rep..

[11] Kardos N. (2020). CRE (Carbapenem Resistant Enterobacteriaceae) and the Globalization of Antimicrobial Resistance: Problems and Solutions. SunText Rev. Biotechnol..

[12] Shahin N.P., Majid E., Amin T.B.A., Bita B. (2019). Host characteristics and virulence typing of *Escherichia coli* isolated from diabetic patients. Gene Rep..

[13] Yousefi B., Abdolshahi A., Dadashpour M., Pahlevan D., Ghaffari H., Eslami M. (2023). Evaluation of genes involved in the binding and invasion of Klebsiella pneumoniae including FimH-1, EntB, IutA, RmpA and Cnf-1 genes in patients with urinary tract infection. Rev. Res. Med. Microbiol..

[14] Peerayeh S.N., Eslami M., Memariani M., Siadat S.D. (2013). High prevalence of blaCTX-M-1 group extended-spectrum β-lactamase genes in *Escherichia coli* isolates from Tehran. Jundishapur J. Microbiol..

[15] Peerayeh S.N., Rostami E., Eslami M., Rezaee M.A. (2016). High frequency of extended-spectrum β-lactamase-producing Klebsiella pneumoniae and *Escherichia coli* isolates from male patients’ Urine. Arch. Clin. Infect. Dis..

[16] Yigit H., Queenan A.M., Anderson G.J., Domenech-Sanchez A., Biddle J.W., Steward C.D., Alberti S., Bush K., Tenover F.C. (2001). Novel carbapenem-hydrolyzing β-lactamase, KPC-1, from a carbapenem-resistant strain of Klebsiella pneumoniae. Antimicrob. Agents Chemother..

[17] Vakili M., Goli H., Javidnia J., Alipour T., Eslami M. (2024). Genetic Diversity and Antibiotic Resistance Patterns of *Escherichia coli* Isolates Causing Septicemia: A Phylogenetic Typing and PFGE Analysis. Diagn. Microbiol. Infect. Dis..

[18] Dubois D., Delmas J., Cady A., Robin F., Sivignon A., Oswald E., Bonnet R. (2010). Cyclomodulins in urosepsis strains of *Escherichia coli*. J. Clin. Microbiol..

[19] Zhu Y., Dong W., Ma J., Yuan L., Hejair H.M., Pan Z., Liu G., Yao H. (2017). Characterization and virulence clustering analysis of extraintestinal pathogenic *Escherichia coli* isolated from swine in China. BMC Vet. Res..

[20] Nagarjuna D., Dhanda R., Gaind R., Yadav M. (2015). tcpC as a prospective new virulence marker in blood *Escherichia coli* isolates from sepsis patients admitted to the intensive care unit. New Microbes New Infect..

[21] Subedi M., Luitel H., Devkota B., Bhattarai R.K., Phuyal S., Panthi P., Shrestha A., Chaudhary D.K. (2018). Antibiotic resistance pattern and virulence genes content in avian pathogenic *Escherichia coli* (APEC) from broiler chickens in Chitwan, Nepal. BMC Vet. Res..

[22] White A., Sibley K., Sibley C., Wasmuth J., Schaefer R., Surette M., Edge T., Neumann N. (2011). Intergenic sequence comparison of *Escherichia coli* isolates reveals lifestyle adaptations but not host specificity. Appl. Environ. Microbiol..

[23] Tapader R., Chatterjee S., Singh A., Dayma P., Haldar S., Pal A., Basu S. (2014). The high prevalence of serine protease autotransporters of Enterobacteriaceae (SPATEs) in *Escherichia coli* causing neonatal septicemia. Eur. J. Clin. Microbiol. Infect. Dis..

[24] Khaled E., Iqbal A. (2022). Antimicrobial Resistance, Virulence Factor-Encoding Genes, and Biofilm-Forming Ability of Community-Associated Uropathogenic *Escherichia coli* in Western Saudi Arabia. Pol. J. Microbiol..

[25] Dhanji H., Doumith M., Clermont O., Denamur E., Hope R., Livermore D.M., Woodford N. (2010). Real-time PCR for detection of the O25b-ST131 clone of *Escherichia coli* and its CTX-M-15-like extended-spectrum β-lactamases. Int. J. Antimicrob. Agents.

[26] Ikuta N., de Oliveira Solla Sobral F., Lehmann F.K.M., da Silveira V.P., de Carli S., Casanova Y.S., Celmer Á.J., Fonseca A.S.K., Lunge V.R. (2014). Taqman real-time PCR assays for rapid detection of avian pathogenic *Escherichia coli* isolates. Avian Dis..

[27] Hou Z., Fink R., Black E., Sugawara M., Zhang Z., Diez-Gonzalez F., Sadowsky M. (2012). Gene expression profiling of *Escherichia coli* in response to interactions with the lettuce rhizosphere. J. Appl. Microbiol..

[28] Navasa N., Rodríguez-Aparicio L.B., Ferrero M.Á., Moteagudo-Mera A., Martínez-Blanco H. (2011). Growth temperature regulation of some genes that define the superficial capsular carbohydrate composition of *Escherichia coli* K92. FEMS Microbiol. Lett..

[29] Moirongo R.M., Lorenz E., Ntinginya N.E., Dekker D., Fernandes J., Held J., Lamshöft M., Schaumburg F., Mangu C., Sudi L. (2020). Regional variation of extended-spectrum beta-lactamase (ESBL)-producing Enterobacterales, fluoroquinolone-resistant Salmonella enterica and methicillin-resistant Staphylococcus aureus among febrile patients in sub-Saharan Africa. Front. Microbiol..

[30] Hijazi K., Joshi C., Gould I.M. (2019). Challenges and opportunities for antimicrobial stewardship in resource-rich and resource-limited countries. Expert Rev. Anti-Infect. Ther..

[31] Wu T., Fu Y., Guo S., Shi Y., Zhang Y., Fan Z., Yang B., Ding B., Liao Y. (2024). Self-assembly multifunctional DNA tetrahedron for efficient elimination of antibiotic-resistant bacteria. Aggregate.

[32] Damlin A. (2020). Responsible Antibiotic Use and Diagnostic Challenges in Infectious Diseases: Studies in a Resource-Limited Setting and a High-Income Setting.

[33] Hawkey J., Wyres K.L., Judd L.M., Harshegyi T., Blakeway L., Wick R.R., Jenney A.W., Holt K.E. (2022). ESBL plasmids in Klebsiella pneumoniae: Diversity, transmission and contribution to infection burden in the hospital setting. Genome Med..

[34] Pitout J.D. (2010). Infections with extended-spectrum β-lactamase-producing Enterobacteriaceae: Changing epidemiology and drug treatment choices. Drugs.

[35] Oguadinma I.C., Mishra A., Juneja V.K., Dev Kumar G. (2022). Antibiotic Resistance Influences Growth Rates and Cross-Tolerance to Lactic Acid in *Escherichia coli* O157:H7 H1730. Foodborne Pathog. Dis..

[36] Zhang W., Chen X., Sun W., Nie T., Quanquin N., Sun Y. (2020). *Escherichia coli* Increases its ATP Concentration in Weakly Acidic Environments Principally through the Glycolytic Pathway. Genes.

[37] Zeng M., Zou Y., Shi Z., Wang J., Yang Y., Bai Y., Ping A., Zhang P., Chen Y., Tao H. (2024). A broad-spectrum broth rapidly and completely repairing the sublethal injuries of *Escherichia coli* caused by freezing and lactic acid alone or in combination for accurate enumeration. LWT.

[38] Fontoura I., Veriato T.S., Raniero L.J., Castilho M.L. (2023). Analysis of Capped Silver Nanoparticles Combined with Imipenem against Different Susceptibility Profiles of Klebsiella pneumoniae. Antibiotics.

[39] Zhou W., Du Y., Li X., Yao C. (2020). Lipoic acid modified antimicrobial peptide with enhanced antimicrobial properties. Bioorg. Med. Chem..

[40] Sun Y., Zhang W., Ma J., Pang H., Wang H. (2017). Overproduction of α-Lipoic Acid by Gene Manipulated *Escherichia coli*. PLoS ONE.

